# Specific Therapy for Transthyretin Cardiac Amyloidosis: A Systematic Literature Review and Evidence‐Based Recommendations

**DOI:** 10.1161/JAHA.120.016614

**Published:** 2020-09-24

**Authors:** Nuno Marques, Olga Azevedo, Ana Rita Almeida, Dina Bento, Inês Cruz, Emanuel Correia, Carolina Lourenço, Luís Rocha Lopes

**Affiliations:** ^1^ Algarve Biomedical Center Algarve Portugal; ^2^ Biomedical and Medical Department Algarve University Algarve Portugal; ^3^ Cardiology Department Centro Hospitalar Universitário do Algarve Algarve Portugal; ^4^ Cardiology Department Hospital Senhora da Oliveira Guimarães Portugal; ^5^ Life and Health Sciences Research Institute (ICVS) School of Medicine University of Minho Braga Portugal; ^6^ ICVS/3Bs PT Government Associate Laboratory Braga/Guimarães Portugal; ^7^ Cardiology Department Hospital Garcia de Orta Almada Portugal; ^8^ Cardiology Department Centro Hospitalar Tondela Viseu Viseu Portugal; ^9^ Cardiology Department Centro Hospitalar Universitário de Coimbra Coimbra Portugal; ^10^ St. Bartholomew’s Hospital‐Barts Heart Centre Barts Health NHS Trust London United Kingdom; ^11^ Centre for Heart Muscle Disease Institute of Cardiovascular Science University College of London United Kingdom; ^12^ Centro Cardiovacular Universidade Lisboa Lisboa Portugal

**Keywords:** amyloid, cardiac amyloidosis, therapy, transthyretin, transthyretin‐related amyloidosis, Mortality/Survival, Quality and Outcomes, Statements and Guidelines, Cardiomyopathy

## Abstract

**Background:**

The emergence of specific therapies for transthyretin cardiac amyloidosis (CA) warrants the need for a systematic review of the literature.

**Methods and Results:**

A systematic review of the literature was conducted according to Preferred Reporting Items for Systematic Reviews and Meta‐Analyses (PRISMA) guidelines. A systematic search was performed on MEDLINE, PubMed, and Embase databases on November 29, 2019. Studies were selected based on the following predefined eligibility criteria: English‐language randomized controlled trials (RCTs), non‐RCTs, or observational studies, which included adult patients with variant/wild‐type transthyretin‐CA, assessed specific therapies for transthyretin‐CA, and reported cardiovascular outcomes. Relevant data were extracted to a predefined template. Quality assessment was based on National Institute for Health and Care Excellence recommendations (RCTs) or a checklist by Downs and Black (non‐RCTs). From 1203 records, 24 publications were selected, describing 4 RCTs (6 publications) and 16 non‐RCTs (18 publications). Tafamidis was shown to significantly improve all‐cause mortality and cardiovascular hospitalizations and reduce worsening in 6‐minute walk test, Kansas City Cardiomyopathy Questionnaire—Overall Summary score, and NT‐proBNP (N‐terminal pro‐B‐type natriuretic peptide) in variant/wild‐type transthyretin‐CA. Patisiran showed promising results in a subgroup analysis of patients with variant transthyretin‐CA, which have to be confirmed in RCTs. Inotersen showed conflicting results on cardiac imaging parameters. The one study on AG10 had only a 1‐month duration and cardiovascular end points were exploratory and limited to cardiac biomarkers. Limited evidence from noncomparative single‐arm small non‐RCTs existed for diflunisal, epigallocatechin‐3‐gallate (green tea extract), and doxycycline+tauroursodeoxycholic acid/ursodeoxycholic acid.

**Conclusions:**

This systematic review of the literature supports the use of tafamidis in wild‐type and variant transthyretin‐CA. Novel therapeutic targets including transthyretin gene silencers are currently under investigation.

**Nonstandard Abbreviations and Acronyms**
ATTR: transthyretin‐related amyloidosis
ATTRv: variant transthyretin‐related amyloidosis
ATTRwt: wild‐type transthyretin‐related amyloidosis
CA: cardiac amyloidosis
EMA: European Medicines Agency
FDA: Food and Drug Administration
KCCQ‐OS: Kansas City Cardiomyopathy Questionnaire—Overall Summary
NYHA: New York Heart Association
TRACS: Transthyretin Amyloidosis Cardiac Study
TUDCA: tauroursodeoxycholic acid
UDCA: ursodeoxycholic acid

**Clinical Perspective**

**What Is New?**

- As novel therapies emerge in the field of transthyretin‐related amyloidosis, this systematic review of the literature examines and presents the existing evidence regarding specific therapies for transthyretin cardiac amyloidosis.
- This systematic review supports the use of tafamidis in patients with transthyretin cardiac amyloidosis, either variant transthyretin‐related amyloidosis or wild‐type transthyretin‐related amyloidosis, but does not support the use of diflunisal, AG10, doxycycline, or epigallocatechin‐3‐gallate in these patients.

**What Are the Clinical Implications?**

- Novel therapies, including transthyretin gene silencers, are currently under investigation in dedicated randomized controlled trials.

Transthyretin‐related amyloidosis (ATTR) results from the tissue deposition of amyloid fibers that are composed of transthyretin, a protein that is mainly produced by the liver and acts as a carrier of thyroxine and retinol‐binding protein bound to retinol.^1^ Fibrillogenesis requires the dissociation of transthyretin tetramers into misfolded monomers that self‐assemble in soluble oligomeric species. Oligomers aggregate into protofibrils and finally mature amyloid fibers, which then deposit within tissues leading to the development of ATTR.^1^ ATTR can result from the deposition of either variant ATTR (ATTRv) or wild‐type ATTR (ATTRwt).^2^

ATTRv is an autosomal dominant disease and >120 pathogenic variants, mostly missense, have been described in the transthyretin gene. The phenotype is variable, ranging from pure polyneuropathy to selective cardiac involvement.^2^ Accurate statistics on the prevalence of ATTRv are difficult to obtain. Among Americans of European descent, the estimated incidence of ATTRv is 0.4 per million people per year; however, this condition is believed to be more common among people with African ancestry and in specific geographic areas, such as northern Portugal, Sweden, Japan, or some regions of West Africa.^2^ In Portugal, endemic ATTRv is caused by the Val30Met mutation and familial amyloidotic polyneuropathy is the predominant feature of the phenotype.

ATTRwt is characteristically associated with almost exclusive cardiac involvement, with carpal tunnel syndrome being one of the few extracardiac red flags. The true prevalence of ATTRwt is unknown, but it may be relatively high compared with the prevalence of ATTRv.^3^ It affects the elderly and, in autopsy studies, nearly 25% of the hearts of individuals aged 80 years or older contained wild‐type transthyretin fibrils, regardless of the presence of symptoms.^3^ Studies using nonbiopsy approaches to diagnose ATTRwt reported prevalence rates of 16% among patients undergoing percutaneous aortic valve replacement for severe aortic stenosis, 13% among patients with heart failure with preserved ejection fraction, 5% among patients with presumed hypertrophic cardiomyopathy, and 7% to 8% among patients with carpal tunnel syndrome on biopsy of the tenosynovial tissue. Furthermore, 1% to 3% of individuals older than 75 years showed myocardial retention of diphosphono‐1,2‐propanodicarboxylic acid, which is indicative of TTR‐related cardiac amyloidosis (CA).^3^

Cardiac involvement is common in ATTR and is associated with a particularly poor life expectancy of 2 to 6 years after diagnosis. Transthyretin CA is a progressive disorder and patients ultimately develop heart failure, dysrhythmias, and cardiac conduction disturbances, which result in decreased functional capacity, diminished quality of life, and eventually death.^3^ Moreover, treatment of transthyretin CA was, until recently, limited to the treatment of symptoms and complications.

However, the emergence of new therapeutic options and the results of recent randomized controlled trials in patients with transthyretin CA warrant the need for a systematic review of the literature.

The aim of the systematic review was to identify and synthesize the available evidence on specific therapies for transthyretin CA and, based on this, to provide evidence‐based recommendations for the use of these therapies. This systematic review will focus on specific therapies targeting the inhibition of transthyretin synthesis (inotersen or patisiran); tetramer stabilization (diflunisal, tafamidis, or AG10); inhibition of oligomer aggregation and disruption (epigallocatechin‐3‐gallate); and degradation and reabsorption of amyloid fibers (doxycycline‐tauroursodeoxycholic [TUDCA] acid or doxycycline‐ursodeoxycholic acid [UDCA]).^4^

## Methods

The authors declare that all supporting data are available within the article.

This systematic review of the literature was conducted using the methodology suggested by Preferred Reporting Items for Systematic Reviews and Meta‐Analyses (PRISMA) guidelines.^5^ It was conducted in 3 stages: a comprehensive and systematic search of the published literature to identify all potentially relevant studies; systematic selection of relevant studies based on explicit inclusion and exclusion criteria; and extraction of relevant data from eligible studies to assess the effects of therapy in patients with transthyretin CA.

### Search Strategy

A systematic search was performed on November 29, 2019, in the following databases: MEDLINE, PubMed, and Embase. The search was performed in the English‐language literature using the following keywords: “transthyretin amyloidosis treatment”; “transthyretin amyloidosis therapy”; “AG10 transthyretin amyloidosis”; “diflunisal transthyretin amyloidosis”; “tafamidis”; “oligonucleotides transthyretin amyloidosis”; “inotersen”; “patisiran”; “green tea transthyretin amyloidosis”; and “doxycycline transthyretin amyloidosis.” A deduplication step was performed to remove studies that overlapped among the databases.

### Selection Criteria

To be included in this review, studies had to meet the predefined eligibility criteria listed in Table 1. In summary, studies included in this review were RCTs, non‐RCTs, or observational studies, including adult patients diagnosed with transthyretin CA, assessing at least 1 of the specific therapies for transthyretin CA and reporting cardiovascular outcomes (Table 1).

**Table 1:** Inclusion and Exclusion Criteria of the Studies for Systematic Review

Category	Inclusion Criteria	Exclusion Criteria
Population	Patients with transthyretin CA Familial or mutant/variant transthyretin CA Wild‐type transthyretin CA Any ethnicity Age ≥18 y	Healthy volunteers only Pediatric population (<18 y) Disease other than transthyretin CA
Interventions	Studies assessing specific therapies for ATTR, namely: AG10 Diflunisal Tafamidis Inotersen Patisiran Doxycycline plus tauroursodeoxycholic acid or ursodeoxycholic acid Green tea extract (epigallocatechin‐3‐gallate)	Nonspecific therapies for ATTR such as diuretics and other nondisease‐modifying therapies. Liver transplantation Studies assessing interventions not included on the list
Comparators	No restrictions	None
Outcomes	Cardiovascular outcomes ◦ Biomarkers ◦ ECG or Holter parameters ◦ Imaging parameters ◦ Functional tests ◦ Quality of life ◦ Arrhythmias ◦ Hospital admissions ◦ Death	Only noncardiovascular outcomes
Study design	RCTs irrespective of blinding status Non‐RCTs Observational studies Single‐arm studies Cohort studies (both prospective and retrospective) Systematic reviews and meta‐analyses of RCTs*/non-RCTs*	Case reports, case series Pharmacokinetic and economic studies Preclinical studies Reviews, letters, and comment articles
Language	English	Other than English

ATTR indicates transthyretin‐related amyloidosis; CA, cardiac amyloidosis; and RCT, randomized controlled trials.

a Systematic reviews and meta‐analyses of RCTs and non‐RCTs were included and flagged. Bibliographies of these systematic reviews were screened to check whether literature searches missed any potentially relevant studies.

Primary screening was performed by 2 authors who independently reviewed each reference (title and abstract) identified by the literature search, applied the inclusion and exclusion criteria listed in Table 1, and decided on whether to include or exclude the publication at that stage. Disagreement regarding the inclusion of studies was solved by a third author. The full‐text articles were obtained for potentially relevant studies identified by primary screening of titles and abstracts. These were independently reviewed by 2 reviewers against each eligibility criteria. Disagreement regarding the inclusion of studies was solved by a third author.

### Data Extraction

The data regarding the cardiovascular outcomes of patients under specific therapies for transthyretin CA were extracted. Safety data on these therapies were also extracted.

The data extraction process was performed by one author to a predefined extraction data template (Tables 2 and 3) and independently checked for errors against the original study report by another author.

**Table 2:** RCTs Included

Study/Reference	Study Phase	Blinding	Study Duration, mo	Treatment	No. of Patients	Mean Age, y	ATTR Type, n	Primary Outcome	Results on Cardiovascular and Safety Outcomes
Inotersen									
NEURO‐TTR Benson et al, 2018^6^	Phase III	Double‐blind	16.5	Inotersen 300 mg	N=112	59.0	ATTRv N=112 CA N=75	mNIS+7 Norfolk QoL‐DN	CA subpopulation Echocardiographic measures IVS thickness (mm) Inotersen vs placebo −0.57 (95% CI, −1.16 to 0.45), *P*=0.270 LVPW thickness (mm) Inotersen vs placebo −0.26 (95% CI, −1.32 to 0.80), *P*=0.621 LVEF (%) Inotersen vs placebo −1.99 (95% CI, −5.49 to 1.50), *P*=0.260 LV mass (g) Inotersen vs placebo −2.8 (95% CI, −22.1 to 16.4), *P*=0.768 E/Em lateral ratio Inotersen vs placebo −1.41 (95% CI, −4.22 to 1.39), *P*=0.317 GLS (%) Inotersen vs placebo +0.20 (95% CI, −1.17 to 1.56), *P*=0.771 Global study population safety outcomes AE (%) Placebo=100% Inotersen=99% Serious AE (%) Placebo=22% Inotersen=32% Study discontinuation because of an AE (%) Placebo=2% Inotersen=14% Mortality caused by an AE (%) Placebo=0% Inotersen=1%
Placebo				N=60	59.5	ATTRv N=60 CA N=30			
Patisiran									
APOLLO Adams et al, 2018^7^ Solomon et al, 2019^8^ Minamisawa et al, 2019^9^	Phase III	Double blind	18	Patisiran 0.3 mg/kg	N=148	62	ATTRv N=148 CA N=90	mNIS+7	CA subpopulation All‐cause hospitalization+mortality Patisiran vs placebo HR, 0.49 (95% CI, 0.30–0.79) Cardiac hospitalizations+mortality Patisiran vs placebo HR, 0.54 (95% CI, 0.25–1.16) NT‐proBNP (ratio of fold‐change) Patisiran vs placebo +0.45 (95% CI, 0.34–0.59), *P*<0.001 10‐m walk test (m/s) Patisiran vs placebo +0.35 (95% CI, 0.24–0.47) Echocardiographic measures LVW thickness (least‐squares mean) Patisiran vs placebo −0.9 (±0.4), *P*=0.017 LVEF (%) Patisiran vs placebo +0.43 (±1.57), *P*=0.785 Cardiac output (L/min) Patisiran vs placebo +0.38 (±0.19), *P*=0.044 LVEDV (mL) Patisiran vs placebo +8.3 (±3.9), *P*=0.036 LVESV (mL) Patisiran vs placebo +2.7 (±2.1), *P*=0.211 Left atrial volume (mL) Patisiran vs placebo −1.0 (±2.7), *P*=0.731 LV mass (g) Patisiran vs placebo −15.8 (±10.9), *P*=0.150 GLS (%) Patisiran vs placebo −1.4 (95% CI, −2.5 to − 0.3), *P*=0.020 Global study population safety outcomes AE (%) Placebo=97% Patisiran=97% Serious AE (%) Placebo=40% Patisiran=36% Study discontinuation because of an AE (%) Placebo=12% Patisiran=5% Mortality caused by an AE (%) Placebo=0% Patisiran=0%
Placebo				N=77	63	ATTRv N=77 CA N=36			
Tafamidis									
ATTR‐ACT Maurer et al, 2018^10^	Phase III	Double‐blind	30	Tafamidis 80 mg 20 mg	N=264	74.5	ATTRv N=63 ATTRwt N=201	All‐cause mortality Cardiovascular hospitalizations	All‐cause mortality Tafamidis vs placebo HR, 0.70 (95% CI, 0.51–0.96) Cardiac hospitalizations Tafamidis vs placebo HR, 0.68 (95% CI, 0.56–0.81) 6‐min walk test (m) Tafamidis vs placebo +75.7 (±9.2), *P*<0.001 KCCQ‐OS score Tafamidis vs placebo +13.7 (±2.1), *P*<0.001 NT‐proBNP (pg/mL) Tafamidis vs placebo −2180 (95% CI, −3326 to −1035), *P*<0.001 Echocardiographic measures IVS thickness (mm) Tafamidis vs placebo −0.44 (95% CI, −1.11 to 0.23) LVPW thickness (mm) Tafamidis vs placebo −0.27 (95% CI, −1.55 to 1.01) LVEF (%) Tafamidis vs placebo +1.51 (95% CI, −0.57 to 3.60) LV stroke volume (mL) Tafamidis vs placebo +6.28 (95% CI, 1.96–10.59) Circumferential global strain (%) Tafamidis vs placebo −2.67 (95% CI, −4.20 to − 1.15) Radial global strain (%) Tafamidis vs placebo +3.53 (95% CI, 1.00–6.06) GLS (%) Tafamidis vs placebo −0.70 (95% CI, −1.43 to 0.02) Safety outcomes AE (%) Placebo=98.9% Tafamidis=98.5% Serious AE (%) Placebo=79.1% Tafamidis=75.4% Study discontinuation because of an AE (%) Placebo=28.8% Tafamidis=21.2% Mortality caused by an AE (%) Placebo=0% Tafamidis=0%
				Placebo	N=177	74.1	ATTRv N=43 ATTRwt N=134		
AG10									
NCT03458130 Judge et al, 2019^11^	Phase II	Double‐blind	1	AG10 400 mg 800 mg	N=32 N=16 N=16	73.8 75.4	ATTRv N=11 ATTRwt N=21	Safety and tolerability	NT‐proBNP (% change from baseline) Placebo=−4±36 AG10 400=+3±33 AG10 800=−14±16 No statistical test applied Troponin I (% change from baseline) Placebo=−7±15 AG10 400=−4±22 AG10 800=0±17 No statistical test applied Safety outcomes AE (%) Placebo=88% AG10 400=63% AG10 800=60% Serious AE (%) Placebo=12% AG10 400=6% AG10 800=0% Study discontinuation because of an AE (%) Placebo=0% AG10 400=0% AG10 800=0%
				Placebo	N=17	73.2	ATTRv N=3 ATTRwt N=14		

AE indicates adverse event; ATTR, transthyretin‐related amyloidosis; ATTRv, variant transthyretin‐related amyloidosis; ATTRwt, wild‐type transthyretin‐related amyloidosis; CA, cardiac amyloidosis; GLS, global longitudinal strain; HR, hazard ratio; IVS, interventricular septum; KCCQ‐OS, Kansas City Cardiomyopathy Questionnaire—Overall Summary; LV, left ventricular; LVEDV, left ventricular end‐diastolic volume; LVEF, left ventricular ejection fraction; LVESV, left ventricular end‐systolic volume; LVPW, left ventricular posterior wall; LVW, left ventricular wall; mNIS, Modified Neuropathy Impairment Score; NT‐proBNP, N‐terminal pro‐B‐type natriuretic peptide; QoL‐DN, Quality of Life‐Diabetic Neuropathy; and RCT, randomized controlled trial.

**Table 3:** List of Non‐RCTs Included

Study/Reference	Study Design	Study Setting	Study Duration, mo	Treatments	No. of Patients	Mean Age, y	ATTR Type	Primary Outcome	Results on Cardiovascular Outcomes
Inotersen									
Benson et al, 2017^12^ Dasgupta et al, 2020^13^	Prospective	Single‐center	36	Inotersen 300 mg SC weekly	N=33	63.4	ATTRv N=10 ATTRwt N=23	Changes in cardiac parameters	Echocardiographic parameter IVS thickness (mm) (vs baseline) 12 mo 0.0 24 mo 1.4 36 mo 3.0 *P*=0.019 LV mass (g) (mo 12 vs baseline) N=15 ATTRv 363±27 vs 352±28 ATTRwt 507±29 vs 470±40 GLS (mo 12 vs baseline) (N=15) ATTRv 12.7 vs 13.6 ATTRwt 10.2 vs 8.6 GLS (mo 36 vs baseline) +0.3±0.9 MRI measures LV mass (%) 12 mo vs baseline 0.54% 24 mo vs baseline 8.5% 36 mo vs baseline 11.5% 6‐min walk test (m) (vs baseline) 12 mo +10.6 24 mo +22.2 36 mo +16.5 *P*=NS BNP (pg/mL) (vs baseline) 12 mo 321.7 vs 353.8 24 mo 319.4 vs 353.8 36 mo 260.8 vs 353.8 *P*=NS NYHA class (mo 12 vs baseline) (N=15) 6 patients improved 6 patients stabilized 3 patients worsened Safety outcomes One patient discontinued therapy because of an AE There was an 11% decrease in platelet count in 15 patients (45%) The other safety parameters remained stable
Patisiran									
Adams et al, 2017^14^	Prospective	Multicenter	24	Patisiran 0.3 g/kg IV every 3 wk	N=27	64.0	ATTRv N=27 Cardiomyopathy N=11	Safety and tolerability	CA subpopulation (24 mo vs baseline) NT‐proBNP (ng/L) −49.6±170.8 Troponin I (ng/mL) −0.1±0.1 Echocardiographic measures LV mass (g) −16.7±11.7 LV wall thickness (mm) −0.8±1 LVEF (%) +0.6±1.4 GLS (%) +0.9±0.9 10‐min walk test (m/s) +0.03±0.05 Safety outcomes 26% patients with a serious AE One patient discontinued therapy because of an AE
Diflunisal									
Castaño et al, 2012^15^	Prospective	Single‐center	10.8	Diflunisal 250 mg bid	N=13	69	ATTRv N=6 ATTRwt N=7	Safety and efficacy	BNP (pg/mL) Baseline=388±92 Follow‐up=481±102 *P*=0.52 Troponin I (ng/mL) Baseline=0.04±0.01 Follow‐up=0.07±0.02 *P*=0.08 Echocardiographic measures IVS thickness (mm) Baseline=18±1 Follow‐up=16±1 *P*=0.25 LVPW thickness (mm) Baseline=16±1 Follow‐up=15±2 *P*=0.50 LV mass (g/m^2^) Baseline=384±37 Follow‐up=331±65 *P*=0.36 LVEF (%) Baseline=50±3 Follow‐up=48±6 *P*=0.61 LVEDD (mm) Baseline=44±1 Follow‐up=45±1 *P*=0.33 LA diameter (mm) Baseline=46±2 Follow‐up=45±2 *P*=0.80 Safety outcomes One patient discontinued therapy because of an AE Well tolerated from hematologic and renal standpoint
Sekijima et al, 2015^16^	Prospective	Single‐center	38.0	Diflunisal 250 mg bid	N=40	60.7	ATTRv N=40 Cardiomyopathy N=34	Safety and efficacy	BNP (% variation from baseline) +11.7±42.5 *P*=0.96 hANP (% variation from baseline) +11.7±32.7 *P*=0.96 Echocardiographic measures IVS+LVPW thickness (mm, variation from baseline) +0.25±1.74 *P*=0.30 LVEF (% variation from baseline) −0.21±3.76 *P*=0.23 Safety outcomes Three patients discontinued therapy because of an AE Deterioration of renal function and thrombocytopenia
Rosenblum et al, 2018 ^17^	Retrospective	Single‐center	22.8	Diflunisal Tafamidis Without Stabilizer	N=120 DiflunisalN=13 TafamidisN=16 Controls N=91	75	ATTRv N=36 ATTRwt N=84	All‐cause mortality or heart transplantation	Mortality or heart transplantation Stabilizer vs without stabilizer HR 0.32 (95% CI, 0.18–0.58), *P*<0.001 Multivariable adjusted HR HR, 0.37 (95% CI, 0.19–0.75), *P*=0.003 The association of type of stabilizer with the outcome did not differ
Wixner et al, 2019^18^	Prospective	Multicenter	24	Diflunisal 500 mg/d	N=54	68	ATTRv N=54	Kumamoto scale	ProBNP (baseline vs Follow‐up (ng/L)) 532 vs 457, *P*=0.19 IVS thickness (baseline vs follow‐up [mm]) 16.5 vs 18; *P*=0.01 Safety 19% of patients discontinued therapy because of an AE Most common side effects were dyspepsia (12%), diarrhea (9%), and increase in creatinine (7%).
TAFAMIDIS									
Merlini et al, 2013^19^ Damy et al, 2015^20^	Prospective Phase II	Multicenter	12	Tafamidis 20 mg QD	N=21	63.1	ATTRv N=21 CA N=12	Transthyretin stability	NT‐proBNP (variation at 12 mo—pg/mL) +75.5 Troponin I (ng/mL) Remained stable ECG abnormalities (12 mo vs baseline) Any 72.2% vs 76.2% Rhythm 33.3% vs 14.3% Conduction 50.0% vs 61.9% Morphology 5.6% vs 4.8% Pathological Q waves 11.1% vs 14.3% Abnormal T wave 11.1% vs 14.3% Holter abnormalities (12 mo vs baseline) Any 72.2% vs 66.7% Atrial fibrillation/flutter 11.8% vs 4.8% Atrial tachycardia 66.7% vs 52.4% NSVT 16.7% vs 38.1% Sustained VT 0% vs 0% Sinus pause 0% vs 4.8% Echocardiographic measures (variation at 12 mo) IVS thickness (mm) +1.0±2.0 LVPW thickness (mm) +0.7±1.7 LA diameter (mm) +1.6±3.5 LV mass (g) +20.9±35.1 LVEF (%) −2.2±5.4 Safety outcomes 86% of patients with an AE 38% patients with a serious AE 14% of patients discontinued therapy because of an AE No statistical test applied
Maurer et al, 2015^21^	Prospective Phase II	Multicenter	12	Tafamidis 20 mg QD	N=31	76.7	ATTRwt N=31	Transthyretin stability	Death 2 patients (6.5%) Cardiovascular hospitalizations 7 patients (22.6%) Worsening heart failure 8 patients (25.8%) NT‐proBNP (variation at 12 mo) (pg/mL) +601±926 Troponin I (variation at 12 mo) (ng/mL) +0.037±0.020; *P*<0.005 Troponin T (variation at 12 mo) (ng/mL) +0.005±0.006; *P*>0.05 6‐min walk test (variation at 12 mo) (ng/mL) +8.9 m Echocardiographic measures (variation at 12 mo) IVS thickness (mm) +1.0 (95% CI, −8.0 to 4.0) LVPW thickness (mm) +1.0 (95% CI, −5.0 to 3.0) RV thickness (mm) 0.0 (95% CI, −4.0 to 5.0) LA diameter (mm) +1.0 (95% CI, −10.0 to 9.0) LVEF (%) −4.0 (95% CI, −29 to 20) Stroke volume (mL) −1.5 (95% CI, −19 to 13) PSAP (mm Hg) +1.0 (95% CI, −19 to 32) E/E´ lateral ratio −1.3 (95% CI, −13.1 to 11.9) E/A ratio +0.6 (95% CI, 0.0–1.4) Isovolumic relaxation time (ms) +7.5 (95% CI, −29 to 28) Holter data (new‐onset) Atrial fibrillation/flutter 44.4% (8 of 18 patients) NSVT 36.4% (4 of 11 patients) Sustained VT 3.8% (1 of 26 patients) Sinus pause 20.8% (5 of 24 patients) Safety outcomes 100% of patients with an AE 42% of patients with a serious AE 3% of patients discontinued therapy because of an AE
Cortese et al, 2016^22^	Prospective	Multicenter	36	Tafamidis	N=61	62.0	ATTRv N=61 CA N=34	Safety Exploratory efficacy on nerve and heart function	Echocardiographic measures IVS thickness (mm) 12 mo vs basal +0.6±1.6 24 mo vs basal +1.05±2.0 Cardiac disease progression By echocardiography 15% at 30 mo By echocardiography+NT‐proBNP 3 patients New development of CA 35% (8/23) NYHA class progression 12 patients Pacemaker implantation 1 patient Safety outcomes 13% of patients with an AE 5% of patients with a serious AE No cases of study discontinuation caused by an AE
Ando et al, 2016^23^	Prospective	Multicenter	19.5	Tafamidis 20 mg QD	N=10	60.1	ATTRv N=10	Transthyretin stability	Echocardiographic measures IVS thickness (mm) Week 52 vs baseline −1.3±2.7 Week 78 vs baseline −3.7±3.7 Stroke volume (mL) Week 52 vs baseline +1.1±5.0 Week 78 vs baseline +6.0±13.0 Safety outcomes 100% of patients with an AE 30% of patients with a serious AE No cases of study discontinuation caused by an AE
Sultan et al, 2017^24^	Comparative analysis of tafamidis study with historical cohort	Multicenter	Tafamidis Fx1B‐201 12 m TRACS 24 m	Tafamidis 20 mg QD No treatment	N=35 Fx1B‐201 N=29 TRACS	Unclear	Treat ATTRwt N=31 ATTRv N=4 Historical cohort ATTRwt N=18 ATTRv N=11	Survival	Time to death (NYHA I or II at baseline) ATTRwt+ATTRv Higher with tafamidis *P*=0.0004 ATTRwt Higher with tafamidis *P*=0.0262
Epigallocatechin‐3‐gallate									
Kristen et al, 2012^25^	Prospective	Single‐center	12	500–700 mg epigallocatechin‐3‐gallate (1.5–2 L green tea or capsules of green tea extract) Mean dose 547±49 mg	N=19	66.3	ATTRv N=10 ATTRwt N=9	Echocardiographic or MRI parameters	Echocardiographic measures Data at 12 mo (14 patients) IVS thickness −6.5%, *P*=0.0081 Decreased in 86% (12/14) Stable in 7% (1/14) Increased in 7% (1/14) LVPW thickness Decreased in 71% (10/14) Increased in 29% (4/14) LV mass Decreased in 64% (9/14) Stable in 14% (2/14) Increased in 21% (3/14) Systolic velocity of lateral mitral annulus Increased in 71% (10/14) Decreased in 29% (4/14) MRI measures Data at 12 mo (9 patients) LV mass (% variation) −12.5%, *P*=0.0039 Decreased or stable in all LVEF (%) Unchanged, *P*=NS Safety outcomes No serious AE
Aus Dem Siepen et al, 2015^26^	Prospective	Single‐center	12	600 mg epigallocatechin‐3‐gallate capsules of green tea extract	N=7	72	ATTRwt N=7	ECV	Data at 12 mo vs baseline (7 patients) Troponin T (μg/L) 26 (20–39) vs 21 (14–36) *P*=NS NT‐proBNP (ng/L) 3920 (2079–6437) vs 2313 (763–5664) *P*=0.11 NYHA class One class I vs 2 class I Three class II vs 2 class II Three class III vs 3 class III *P*=NS Echocardiographic parameters E/A ratio 1.5 (0.96–2.0) vs 1.15 (0.67–1.53) *P*=NS E/E´ ratio 10 (7–21) vs 9 (7–15) *P*=NS ECG findings RBBB 2 vs 2 *P*=NS LBBB 2 vs 2 *P*=NS LAFB 3 vs 3 *P*=NS AF 3 vs 3 *P*=NS CMR parameters LV mass (g) Median decrease—6% 166 (126–207) vs 179 (150–217) *P*=0.046 LVEF (%) 55 (47–64) vs 57 (48–65) *P*=0.3 Native T1 (ms) Median decrease—4% 1080 (970–1120) vs 1110 (1042–1174) *P*=0.031 Post‐contrast T1 (ms) 166 (126–207) vs 179 (150–214) *P*=ns ECV (%) 41.9 (38.4–44.4) vs 42.9 (36.0–47.3) *P*=0.47
Aus Dem Siepen et al, 2015^27^	Prospective	Single‐center	12	600 mg epigallocatechin‐3‐gallate capsules of green tea extract	N=25	71	ATTRwt N=25	Echocardiographic or MRI parameters	NT‐proBNP (ng/L) 3681 vs 3617 Echocardiographic measures 12 mo vs baseline IVS thickness (mm) 18 (14–25) vs 17 (13–28) *P*=NS LVPW thickness (mm) 17 (12–19) vs 16 (11–24) *P*=NS LV mass (g) 335 (228–501) vs 340 (173–523) *P*=NS LVEDD (mm) 42 (33–54) vs 44 (36–53) *P*=NS LVESD (mm) 32 (24–49) vs 33 (23–45) *P*=ns MAPSE (mm) 8 (4–13) vs 10 (5–23) *P*=0.3 TAPSE (mm) 15 (2–20) vs 14 (8–23) *P*=0.4 E/E´ 14 (4–31) vs 11 (7–28) *P*=0.8 MRI measures 12 mo vs baseline (14 patients) LV mass (g) −5.9%,*P*=0.03 180 (85–237) vs 196 (100–247) LVEF (%) 54 (28–71) vs 53 (33–69) *P*=0.75 LVEDV (mL) 162 (102–289) vs 161 (87–278) *P*=NS LVESV (mL) 76 (40–207) vs 69 (40–185) *P*=ns MAPSE (mm) 6 (3–9) vs 7 (3–10) *P*=0.08 TAPSE (mm) 11 (5–16) vs 12 (4–17) *P*=0.28 Safety outcomes No serious AE
Doxycycline									
Obici et al, 2012^28^	Prospective Phase II	Multicenter	12	Doxycycline 100 mg BID TUDCA 250 mg 3 times per d	N=20	64.0	ATTRv N=17 ATTRwt N=3 cardiomyopathy N=17	Response rate <2‐point increase in Neuropathy Impairment Score‐Lower Limb <10% decrease in modified BMI <30% or <300 pg/mL increase in NT‐proBNP	Data at 12 mo (7 patients) NT‐proBNP Increase >30% in 3 patients Stable in 4 patients IVS thickness Stable in 5 patients Decrease >2 mm in 2 patients NYHA class Stable in all patients Safety outcomes No serious AE Treatment was discontinued in 2 patients because of an AE (gastrointestinal AE)
Wixner et al, 2017^29^	Prospective Phase II	Multicenter	18	12 mo Doxycycline 200 mg d/4 wk with 2 wk without doxycycline UDCA 750 mg/d 6 mo Withdrawal	N=28	72	ATTRv N=27 ATTRwt N=1	ProBNP Total Kumamoto score	Data at 12 mo (10 patients) ProBNP Increase at 12 mo *P*<0.005 IVS thickness Stable at 12 mo 13 vs 15 mm, *P*=0.70 Safety outcomes Treatment was discontinued in 14% of patients because of an AE

AE indicates adverse event; AF, atrial fibrillation; ATTR, transthyretin‐related amyloidosis; ATTRv, variant transthyretin‐related amyloidosis; ATTRwt, wild‐type transthyretin‐related amyloidosis; BMI, body mass index; CA, cardiac amyloidosis; ECV, extracellular volume; GLS, global longitudinal strain; IVS, interventricular septum; LA, left atrial; LAFB, left anterior fascicular block; LBBB, left bundle branch block; LV, left ventricular; LVEDD, left ventricular end‐diastolic diameter; LVEDV, left ventricular end‐diastolic volume; LVEF, left ventricular ejection fraction; LVESD, left ventricular end‐systolic diameter; LVESV, left ventricular end‐systolic volume; LVPW, left ventricular posterior wall; MAPSE, mitral annular plane systolic excursion; NSVT, nonsustained ventricular tachycardia; NYHA, New York Heart Association; PASP, pulmonary artery systolic pressure;RBBB, right bundle branch block; RCT, randomized controlled trial; TAPSE, tricuspid annular plane systolic excursion; TRACS, Transthyretin Amyloidosis Cardiac Study; TUDCA, tauroursodeoxycholic acid; UDCA, ursodeoxycholic acid; and VT, ventricular tachycardia.

Multiple publications regarding the same patient population and reporting data for the same intervention were linked together and extracted as a single reference.

### Quality Assessment

**Figure 1:**
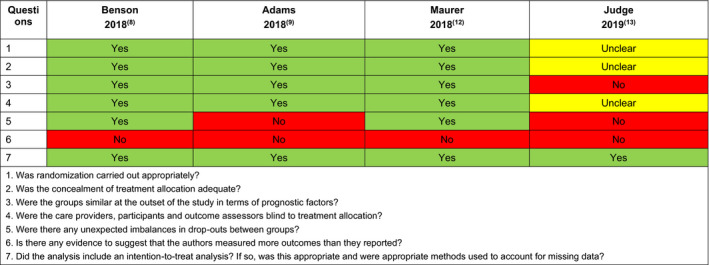
Quality assessment of randomized controlled trials (RCTs).

**Figure 2:**
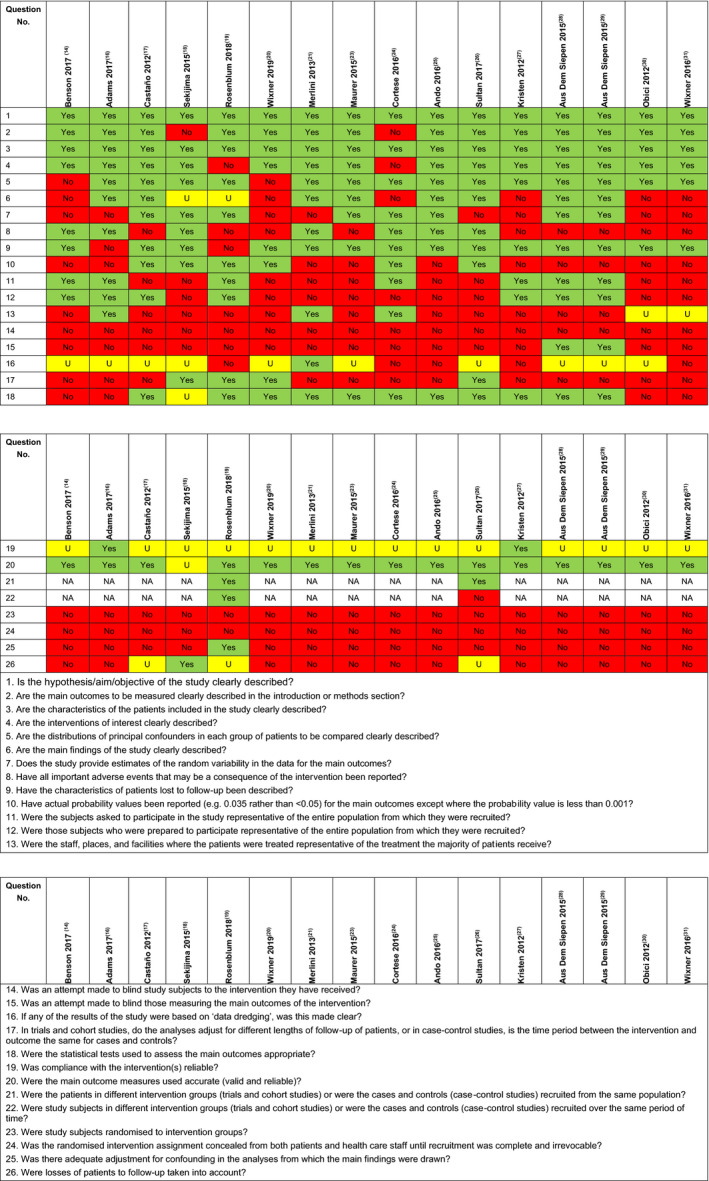
Quality assessment of non‐randomized controlled trials (RCTs). NA indicates not applicable; and U, unable to determine.

Quality assessment of the included RCTs was performed using comprehensive assessment criteria based on recommendations in the National Institute for Health and Care Excellence template^30^ (Figure 1). Quality assessment of the included non‐RCTs was performed using a checklist by Downs and Black^31^ (Figure 2).

## Results

### Literature Selection

**Figure 3:**
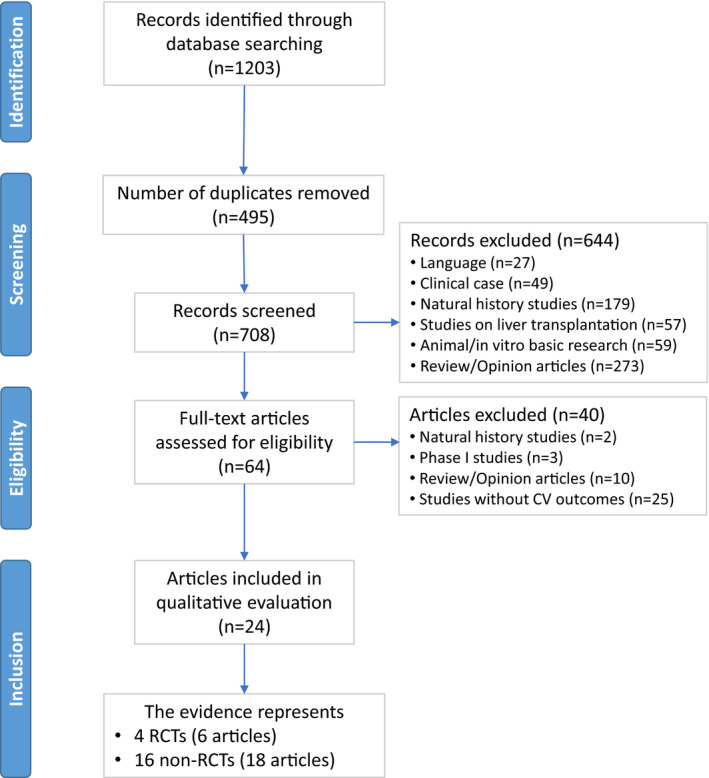
Preferred Reporting Items for Systematic Reviews and Meta‐Analyses (PRISMA) flow diagram. CV indicates cardiovascular; RCT, randomized controlled trial.

Database searches identified 1203 records, of which 495 studies were identified as duplicates and excluded. The screening of the remaining 708 studies led to the exclusion of 644 records, mainly because they were review or opinion articles, natural history studies, or in vitro or animal research studies. The remaining 64 records were further assessed for their eligibility for this review by full‐text screening, which resulted in the additional exclusion of 40 publications, mainly because of the absence of reported cardiovascular outcomes. Relevant data were then extracted from 24 publications, namely 4 RCTs reported in 6 publications and 16 non‐RCTs reported in 18 articles. Figure 3 presents the PRISMA flow diagram of the studies identified in this systematic review of the literature.

### Included Studies and Quality Assessment

A total of 4 RCTs were included in this review. The study characteristics of these trials are presented in Table 2 and their quality assessment in Figure 1.

The 4 RCTs included in this review were the following:

1. a phase III study (NEURO‐TTR ) assessing inotersen versus placebo and including 172 patients, all with ATTRv, 105 of them with CA;
2. a phase III study (APOLLO) assessing patisiran versus placebo and including 225 patients, all with ATTRv, 126 of them with CA^7^, ^8^, ^9^;
3. a phase III study (ATTR‐ACT) assessing tafamidis versus placebo and including 441 patients with CA, 106 with ATTRv and 335 ATTRwt^10^; and
4. a phase II study (NCT03458130) assessing AG10 versus placebo and including 49 patients with CA, 14 with ATTRv and 35 ATTRwt.^11^

A total of 16 non‐RCTs were included in this review. The study characteristics of these trials are presented in Table 3 and their quality assessment in Figure 2.

The 16 non‐RCTs included in this review were the following:

1. one study on inotersen, single‐arm, assessing 33 patients, 10 with ATTRv and 23 with ATTRwt^12^, ^13^;
2. one study on patisiran, single‐arm, assessing 27 patients, all with ATTRv^14^;
3. four studies on diflunisal, including 3 single‐arm and 1 comparative study of stabilizer therapy (diflunisal or tafamidis), assessing a total of 120 patients, with individualized data available from 100 patients with ATTRv and 7 patients with ATTRwt ^15^, ^16^, ^17^, ^18^;
4. five studies on tafamidis, including 4 single‐arm and 1 comparative study of tafamidis with a historical cohort, assessing a total of 127 patients, 96 with ATTRv and 31 ATTRwt^19^, ^20^, ^21^, ^22^, ^23^, ^24^;
5. three studies on epigallocatechin‐3‐gallate (green tea extract), all single‐arm, assessing 51 patients, 10 with ATTRv and 41 with ATTRwt^25^, ^26^, ^27^; and
6. two studies on doxycycline, both single‐arm, assessing 48 patients, 44 with ATTRv and 4 with ATTRwt.^28^, ^29^

### Results of Cardiovascular and Safety Outcomes

The data on the cardiovascular and safety outcomes that were extracted from the studies included in this review are detailed in Tables 2 and 3. In this section, we present the main results of the studies on each specific therapy for transthyretin CA.

#### Inhibition of Transthyretin Synthesis

##### Inotersen

Inotersen was assessed in 1 non‐RCT^12^, ^13^ and 1 RCT.^6^

The non‐RCT was a prospective single‐arm study, with a duration of 36 months, which included 33 patients treated with inotersen, 10 with ATTRv and 23 with ATTRwt, and reported results on cardiovascular biomarkers (BNP), echocardiographic and CMR parameters, and clinical data (6‐minute walk test and New York Heart Association [NYHA] class). The study compared data at the end of the study or at 12‐month follow‐up with baseline data. The interventricular septum thickness measured by echocardiography was lower at study end/12‐month follow‐up than at baseline, but no significant statistical difference was found on BNP, 6‐minute walk test, or other parameters (Table 3).^12^, ^13^

The phase III double‐blind RCT, NEURO‐TTR, included 172 patients with ATTRv, 105 of whom had CA, and compared inotersen with placebo, for a duration of 16.5 months, reporting echocardiographic parameters as exploratory outcomes. In the subset of patients with CA, inotersen did not show a significant effect on the echocardiographic parameters that were evaluated (Table 2).^6^ Moreover, in patients taking inotersen, there was a higher frequency of serious adverse events (placebo 22% versus inotersen 32%) and study discontinuation caused by adverse events (placebo 2% versus inotersen 14%); and one death occurred related to adverse events (placebo 0% versus inotersen 1%) (Table 2). The main serious adverse events associated with inotersen therapy were the occurrence of glomerulonephritis and thrombocytopenia.^6^

##### Patisiran

Patisiran was assessed in 1 non‐RCT^14^ and 1 RCT.^7^, ^8^, ^9^

The non‐RCT was a prospective single‐arm study, with a duration of 24 months, which included 27 patients with ATTRv treated with patisiran, 11 of whom had CA, and reported exploratory data on cardiovascular biomarkers (natriuretic peptide and troponin), echocardiographic parameters, and 10‐meter walk test. In the subgroup of patients with CA, data at 24 months of follow‐up were compared with baseline data, although without applying any statistical test (Table 3).^14^

The phase III double‐blind RCT, APOLLO, comparing patisiran and placebo, with a duration of 18 months, included 225 patients with ATTRv, 126 of whom had CA, and reported data for a prespecified analysis of the subpopulation with CA. Outcomes included mortality and hospitalization, mortality and cardiac hospitalization, 10‐meter walk test, cardiovascular biomarkers (natriuretic peptide), and echocardiographic parameters. This subgroup analysis of patients with CA showed that patisiran, in comparison with placebo, appeared to be associated with lower mortality and hospitalization rate with an hazard ratio (HR) of 0.49 (95% CI, 0.30–0.79), higher velocity in 10‐meter walk test with a mean difference of 0.35 m/s (95% CI, 0.24–0.47), lower NT‐proBNP (N‐terminal pro‐B‐type natriuretic peptide), with a ratio of fold‐change of 0.45 (95% CI, 0.34–0.59), lower left ventricular wall thickness, and a better cardiac output and global longitudinal strain. The results of the other parameters did not reach statistical significance (Table 2).^7^, ^8^, ^9^

No significant safety issues related to patisiran were found in these studies (Table 2 and Table 3).^7^, ^8^, ^9^, ^14^

#### Tetramer Stabilization

##### Diflunisal

Diflunisal was assessed in 4 non‐RCTs, with a small number of patients included in each study (between 13 and 54 patients).^15^, ^16^, ^17^, ^18^ Three of these studies were noncomparative single‐arm prospective treated cohorts, reporting cardiovascular biomarkers (natriuretic peptides and troponin) and echocardiographic parameters as exploratory end points.^15^, ^16^, ^18^ These studies compared data at the end of the study with the baseline data (follow‐up of 10.8–38.0 months) and suggested stabilization of biomarkers and echocardiographic parameters (Table 3).^15^, ^16^, ^18^ The other non‐RCT was a retrospective study comparing patients on stabilizer therapy, diflunisal (n=13), or tafamidis (n=16), with patients without stabilizer therapy.^17^ In this study, the stabilizer therapy was associated with significantly lower all‐cause mortality or heart transplantation, although individualized data for each type of stabilizer was not reported (Table 3).^17^ Notably, the available data for diflunisal refers mainly to patients with ATTRv (n=100) and less to patients with ATTRwt (n=7).

Studies have reported intolerance to diflunisal in some patients, mainly related to gastrointestinal, renal, and hematological adverse events (Table 3).^15^, ^16^, ^17^, ^18^

##### Tafamidis

Tafamidis was assessed in 5 non‐RCTs^19^, ^20^, ^21^, ^22^, ^23^, ^24^ and 1 RCT.^10^

Three of the non‐RCTs were prospective noncomparative single‐arm studies in patients with ATTRv, which included a small number of treated patients (between 10 and 61 patients) and reported cardiovascular outcomes, namely cardiac biomarkers (natriuretic peptides and troponin), ECG and echocardiographic parameters, and, in 1 of the studies, NYHA class progression. These studies compared the data at the end of the study with the baseline data (follow‐up of 12–36 months), and suggested stabilization of biomarkers, ECG and echocardiographic parameters, and NYHA class in patients with ATTRv (Table 3). However, either no statistical test was applied or the outcomes were only exploratory.^19^, ^20^, ^22^, ^23^ One prospective noncomparative single‐arm non‐RCT in patients with ATTRwt, including 31 patients, reported clinical outcomes (death, cardiovascular hospitalizations, worsening heart failure, and 6‐minute walk test), cardiovascular biomarkers (natriuretic peptides and troponin), and echocardiographic and Holter parameters. This study compared data at the end of the study with baseline (follow‐up of 12 months) regarding 6‐minute walk test, cardiovascular biomarkers, and echocardiographic parameters, suggesting stabilization of all of the analyzed parameters with the exception of NT‐proBNP, which showed a mean increment of 601 pg/mL in the 12 months (Table 3). This study also reported the occurrence of clinical events and new‐onset bradyarrhythmias or tachyarrhythmias, although without providing any comparative analysis.^21^

One non‐RCT compared mortality data of a prospective study including 31 patients with ATTRwt and 4 patients with ATTRv treated with tafamidis with an untreated historical cohort from the TRACS (Transthyretin Amyloidosis Cardiac Study) registry, including 18 patients with ATTRwt and 11 patients with ATTRv. This study suggested a significantly higher survival in patients treated with tafamidis, both in the total cohort of patients and in patients with ATTRwt.^24^

The phase III double‐blind RCT, ATTR‐ACT, including 441 patients, 106 with ATTRv and 335 with ATTRwt (76.0%), compared tafamidis 20 or 80 mg with placebo, for a duration of 30 months. It reported data on all‐cause mortality and cardiovascular hospitalizations as the primary outcomes and, also, clinical outcomes (6‐minute walk test and Kansas City Cardiomyopathy Questionnaire—Overall Summary [KCCQ‐OS] score), cardiovascular biomarkers (natriuretic peptide), and echocardiographic parameters as secondary outcomes. This study demonstrated a significant reduction in all‐cause mortality with an HR of 0.70 (95% CI, 0.51–0.96) and cardiovascular hospitalizations with an HR of 0.68 (95% CI, 0.56–0.81) in patients treated with tafamidis. Tafamidis also reduced the decline in 6‐minute walk test and KCCQ‐OS score and was associated with a smaller increase of NT‐proBNP. A smaller decrease of left ventricular stroke volume and less worsening of circumferential and radial global strain were also noted in patients treated with tafamidis, but no effect was detected in the other echocardiographic parameters that were evaluated (Table 2).^10^

The non‐RCTs reported the occurrence of some adverse events,^19^, ^20^, ^21^, ^22^, ^23^ but the RCT ATTR‐ACT has not identified significant safety problems associated with tafamidis therapy in comparison to placebo (Tables 2 and 3).^10^

##### AG10

A phase II double‐blind RCT, with a duration of 1 month, comparing AG10 and placebo and including 49 patients, 14 with ATTRv and 35 with ATTRwt, reported safety outcomes and exploratory data of cardiovascular biomarkers. However, no comparative statistic test was applied (Table 2). In this study there were no safety issues associated with AG10 therapy (Table 2).^11^

#### Inhibition of Oligomer Aggregation and Disruption

##### Epigallocatechin‐3‐gallate (green tea extract)

Epigallocatechin‐3‐gallate (green tea extract) was assessed in 3 non‐RCTs,^25^, ^26^, ^27^ which were noncomparative prospective single‐arm studies, including a total of 51 patients, 10 with ATTRv and 41 with ATTRwt, with a 12‐month follow‐up, but in which data were only available for a small number of patients (from 7 to 25 patients). Cardiovascular outcomes of these studies were cardiac biomarkers (natriuretic peptides and troponin) and ECG, echocardiographic, and CMR parameters. One study (n=14) showed that interventricular septum thickness, measured by echocardiography, was lower at follow‐up than at baseline, but this result was not supported by another study (n=25). In all studies, left ventricular mass, measured by CMR, was also significantly lower at follow‐up than at baseline, although the difference was small. One study reported a significantly lower native T1 time (n=7) at 12 months, although the difference was small and without a significant change in extracellular volume. All of the other analyzed parameters remained stable at 12 months (Table 3).^25^, ^26^, ^27^

These studies have not identified significant safety problems associated with green tea extract therapy (Table 3).^25^, ^26^, ^27^

#### Degradation and Reabsorption of Amyloid Fibers

##### Doxycycline+TUDCA or UDCA

Doxycycline was assessed in 2 non‐RCTs^28^, ^29^ (1 study in association with TUDCA^28^ and 1 study in association with UDCA^29^

The 2 non‐RCTs were noncomparative prospective single‐arm studies, including 48 patients, 44 with ATTRv and 4 with ATTRwt.^28^, ^29^ In the study with doxycycline and TUDCA, a stabilization of natriuretic peptide, interventricular septum thickness, and NYHA class was reported in 7 patients at 12‐month follow‐up.^28^ In the other study with doxycycline and UDCA, interventricular septum thickness remained stable, but proBNP levels were significantly higher at 12 months in 14 patients (Table 3).^29^ Notably, data were available from only 21 patients.^28^, ^29^

Studies have reported intolerance to doxycycline in some patients leading to treatment discontinuation (Table 3).^28^, ^29^

## Discussion

This systematic review of the literature showed a clinical benefit in cardiovascular outcomes of patients with transthyretin CA, either ATTRv or ATTRwt, treated with tafamidis. The potential benefit of patisiran in patients with variant transthyretin CA needs to be confirmed in RCTs. Further studies are needed to evaluate the clinical efficacy and safety of other therapies in patients with transthyretin CA.

This systematic review allows us to recommend, with a high degree of certainty, the use of tafamidis in transthyretin CA, both ATTRv and ATTRwt (Class of Recommendation I, Level of Evidence B). This recommendation is mainly based on the results of a large and well‐designed phase III RCT, ATTR‐ACT.^10^ In this study, tafamidis significantly improved all‐cause mortality and cardiovascular hospitalizations, reduced the decline in 6‐minute walk test and KCCQ‐OS score, and was associated with a smaller increase of NT‐proBNP, smaller decrease of left ventricular stroke volume, and less worsening of radial and circumferential global strain in patients with CA, caused by ATTRv or ATTRwt. Of note, in the subgroup analysis of ATTR‐ACT, the occurrence of cardiovascular hospitalizations was not significantly different between tafamidis and placebo in the subgroup of patients with ATTRv. However, it should be noted that the study had no statistical power to draw conclusions from the subgroups and the *P* interaction was not significant. In ATTR‐ACT,^10^ tafamidis use was safe. Based on the available evidence, the use of tafamidis was approved in adults with transthyretin CA (ATTRwt and ATTRv) by the Food and Drug Administration (FDA) in 2019 and the European Medicines Agency (EMA) in February 2020.

This systematic review of the literature also suggests that patisiran is a promising drug for patients with variant transthyretin CA.

This hypothesis is mainly based on a subgroup analysis of a phase III double‐blind RCT, APOLLO,^7^, ^8^, ^9^ which suggested that treatment with patisiran in these patients was associated with a reduction in mortality and hospitalization, as well as a reduction in NT‐proBNP and left ventricular wall thickness and less worsening in cardiac output, global longitudinal strain, and 10‐meter walk test. In APOLLO,^7^, ^8^, ^9^ patisiran use was safe, without significant adverse events. The potential benefit of patisiran in patients with variant transthyretin CA is currently under investigation in the APOLLO‐B phase 3 RCT. Moreover, there are no available data so far to support the use of patisiran in patients with CA caused by ATTRwt and results on these patients will also be provided by the ongoing APOLLO‐B trial. Patisiran is currently only approved by the EMA and FDA in adults with ATTRv‐related polyneuropathy.

Further studies will be needed to evaluate the effect of inotersen in patients with transthyretin CA, as data in the only 2 studies were contradictory, with 1 non‐RCT^12^, ^13^ showing an improvement of the exploratory outcome of interventricular septum thickness measured by echocardiography, and 1 RCT, the NEURO‐TTR trial,^6^ showing an absence of significant effect on the echocardiographic parameters in the subgroup of patients with CA caused by ATTRv. Inotersen, in the NEURO‐TTR trial,^6^ was also associated with the occurrence of severe adverse events, namely glomerulonephritis and thrombocytopenia. Inotersen is currently only approved by the EMA and FDA in adults with ATTRv‐related polyneuropathy.

Similarly, the use of diflunisal cannot be recommended at the present time in patients with transthyretin CA, because the studies on diflunisal^15^, ^16^, ^18^ are all noncomparative small non‐RCTs, including almost exclusively patients with ATTRv, with exploratory cardiovascular end points limited to cardiovascular biomarkers and echocardiographic parameters. Furthermore, intolerance to diflunisal has been reported in some patients, mainly related to gastrointestinal, renal, and hematological adverse events. The use of diflunisal in transthyretin CA is not currently approved by the EMA or FDA.

Similarly, AG10 cannot be recommended at the present time for transthyretin CA, as the only study of AG10^11^ had a short duration of 1 month and the cardiovascular end points are merely exploratory and limited to cardiac biomarkers. Accordingly, the use of AG10 in transthyretin CA is not currently approved by the EMA or FDA.

Although a small but significant benefit was found on cardiac MRI parameters such as left ventricular mass and native T1 in patients with transthyretin CA treated with epigallocatechin‐3‐gallate (green tea extract), these results should be interpreted carefully, as they are based on noncomparative single‐arm small non‐RCTs.^25^, ^26^, ^27^ Therefore, the use of epigallocatechin‐3‐gallate cannot be recommended at the present time, in transthyretin CA. The EMA and FDA also did not approve the use of epigallocatechin‐3‐gallate in transthyretin CA.

Similarly, results on doxycycline+TUDCA or UDCA are contradictory and based on 2 noncomparative^28^, ^29^ single‐arm small non‐RCTs. Doxycycline+TUDCA or UDCA are currently not approved by the EMA or FDA in transthyretin CA.

Further studies, preferentially large double‐blinded RCTs, evaluating not only cardiac biomarkers or imaging parameters but also hard clinical end points, such as all‐cause mortality, cardiovascular mortality, and cardiovascular hospitalizations, will be needed to better evaluate the efficacy of patisiran, inotersen, diflunisal, AG10, epigallocatechin‐3‐gallate, and doxycycline in patients with transthyretin CA.

This is an enthusiastic field of investigation. Many randomized, double‐blind, placebo‐controlled phase 3 studies are currently underway and their results may change the paradigm of treatment of ATTR cardiomyopathy in the coming years. APOLLO B is evaluating patisiran in patients with ATTR cardiomyopathy (ATTRwt or ATTRv), considering 6‐minute walk test as the primary end point and death and hospitalization outcomes as secondary end points at 12 months. The HELIOS‐B study is evaluating another RNA interference agent, vutrisiran, with the advantage of a subcutaneous administration every 3 months. This study will also include patients with ATTR cardiomyopathy, either ATTRwt or ATTRv, but the primary end point is the composite outcome of all‐cause mortality and recurrent cardiovascular hospitalizations at 30 months. The CARDIO‐TTRansform trial is evaluating another antisense oligonucleotide, AKCEA‐TTR‐L_RX_, with the advantage of a lower frequency of administration (every 4 weeks) and possibly of side effects compared with inotersen. This study will also include patients with ATTR cardiomyopathy, either ATTRwt or ATTRv, and the primary end point is cardiovascular mortality and clinical events at 120 weeks. The ATTRIBUTE‐CM study is evaluating AG10 in patients with ATTR cardiomyopathy (ATTRwt or ATTRv) and the primary end points are 6‐minute walk test change from baseline at 12 months and all‐cause mortality and cardiovascular hospitalizations at 30 months.

On the other hand, past research has already led to the discontinuation of drugs in this field. The phase 3 ENDEAVOUR study was prematurely terminated after a median of 6.71 months because of a mortality imbalance between revusiran, an RNA interference agent targeting transthyretin production, and placebo (12.9% versus 3.0%) in patients with variant transthyretin CA. Most deaths were adjudicated as cardiovascular caused by heart failure, consistent with the natural history of the disease. Although no clear causative mechanism could be identified, a role for revusiran cannot be excluded and further development of this compound has been discontinued.^32^

Despite the intense ongoing investigation, there is still an evidence gap concerning particular subsets of patients who have been excluded from the studies, namely patients developing TTR CA after liver transplantation, patients with TTR CA presenting with advanced heart failure (NYHA class IV), and elderly patients with ATTRwt (older than 90 years). Studies on these subsets of patients are needed to be able to provide evidence‐based recommendations on the use of these therapies in these patients.

Considering the high cost of some of these therapies, cost‐effectiveness studies should also be performed in the near future. Kazi et al^33^ recently published a cost‐effectiveness study for the use of tafamidis in ATTR cardiomyopathy, highlighting the need to lower the cost of tafamidis to be cost‐effective.

Finally, studies are needed to assess whether the association of therapies with different mechanisms of action could improve the outcome of transthyretin CA.

Precision medicine might guide clinicians in the management of patients with transthyretin CA by providing the best therapy or combination of therapies for each patient, thereby maximizing effectiveness and reducing undesirable effects.

## Conclusions

This systematic review of the literature found and analyzed 4 RCTs and 16 non‐RCTs in a total of 24 studies evaluating specific therapies for transthyretin CA. The available data allow us to recommend, with a high degree of certainty, the use of tafamidis in patients with transthyretin CA, both ATTRv and ATTRwt. Novel therapeutic targets including transthyretin gene silencers are currently under investigation. In addition, the systematic review of the available evidence does not support, at the present time, the use of diflunisal, AG10, doxycycline, or epigallocatechin‐3‐gallate in transthyretin CA.

## Sources of Funding

The Municipality of Loulé supported the publication of this article.

## Disclosures

Dr Azevedo received consulting and speaker fees, such as travel and/or accommodation support for conferences, from Pfizer and Alnylam. Dr Lopes received consulting fees from Pfizer. The remaining authors have no disclosures to report.
